# Reactions of Hydrogen-Passivated Silicon Vacancies in *α*-Quartz with Electron Holes and Hydrogen

**DOI:** 10.3390/nano15020142

**Published:** 2025-01-19

**Authors:** Teofilo Cobos Freire, Jack Strand, Alexander L. Shluger

**Affiliations:** 1 Department of Physics and Astronomy, University College London, Gower Street, London WC1E 6BT, UK; jack.strand.14@ucl.ac.uk; 2 Nanolayers Research Computing Ltd., London NW9 6PL, UK; 3 WPI-Advanced Institute for Materials Research (WPI-AIMR), Tohoku University, 2-1-1 Katahira, Aoba-ku, Sendai 980-8577, Japan

**Keywords:** density functional theory, *α*-quartz, Si vacancies, reactions with hydrogen, charge trapping, device reliability

## Abstract

We used density functional theory with a hybrid functional to investigate the structure and properties of [4H]*_Si_* (hydrogarnet) defects in *α*-quartz as well as the reactions of these defects with electron holes and extra hydrogen atoms and ions. The results demonstrate the depassivation mechanisms of hydrogen-passivated silicon vacancies in *α*-quartz, providing a detailed understanding of their stability, electronic properties, and behaviour in different charge states. While fully hydrogen passivated silicon vacancies are electrically inert, the partial removal of hydrogen atoms activates these defects as hole traps, altering the defect states and influencing the electronic properties of the material. Our calculations of the hydrogen migration mechanisms predict the low energy barriers for H^+^, H^0^, and H^−^, with the lowest barrier of 0.28 eV for neutral hydrogen migration between parallel c-channels and a similar barrier for H^+^ migration along the c-channels. The reactions of electron holes and hydrogen species with [4H]*_Si_* defects lead to the breaking of O–H bonds and the formation of non-bridging oxygen hole centres (NBOHCs) within the Si vacancies. The calculated optical absorption energies of these centres are close to those attributed to individual NBOHCs in glass samples. These findings can be useful for understanding the role of [4H]*_Si_* defects in bulk and nanocrystalline quartz as well as in SiO_2_-based electronic devices.

## 1. Introduction

We investigated the structure and electronic properties of Si vacancies in SiO_2_ α-quartz, the most abundant silica polymorph in nature [1] with wide applications in glassmaking, watches and clocks, electronics, and precision resonators for frequency control in aerospace and in many other areas [2]. Quartz nanocrystals and nanoplates are grown using hydrothermal synthesis for many applications [3,4]. Point defects can be incorporated into quartz during crystallisation under various thermodynamic conditions and as a result of alteration, irradiation, and other external factors [1,2]. Natural quartz grains from the Earth’s crust contain on average 10 wt ppm of water [5], while hydrothermally grown synthetic quartz crystals often have an even higher water content (see, e.g., [6,7]). The water in quartz is present as molecular water in fluid inclusions and bubbles and in point defects where protons compensate for the charges of vacancies or metal impurities [5,7]. In particular, the existence of fully H-passivated Si vacancies, the hydrogarnet [4H]_*Si*_ defects, is well established in quartz minerals [1,5]. This complex defect can be considered as four protons passivating a Si vacancy, which has a formal charge of –4. This results in the formation of four O-H bonds or Si-O-H (silanol) groups pointing inside the vacancy. The mechanism of its formation in quartz and other minerals is complicated and depends on the growth conditions and exposure to water [8].

The presence of OH defects in quartz is mainly identified by measuring the infrared spectroscopy of the bands related to OH stretching vibrational modes [5,9], aided by theoretical modelling [10]. Theoretical calculations using density functional theory (DFT) have been used to predict the structure and properties of hydrogarnet [4H]_*Si*_ defects in α-quartz [11,12,13], amorphous silica [14], and zeolites [15,16]. However, only neutral charge states of the fully H passivated defect have been considered. Reactions with electron holes and hydrogen species can change the structure and properties of [4H]_*Si*_ defects. In particular, the exposure of samples to ionising radiation creates electron holes. The latter can be trapped by [4H]_*Si*_ defects in quartz, as demonstrated by electron paramagnetic resonance (EPR) in [17]. Due to wide applications, radiation effects in quartz have been studied extensively [18,19,20], but the mechanisms of hole-induced reactions with defects are poorly understood. A better understanding of the radiation-induced defect processes in quartz could also inform radiation damage studies of silicate glasses used in nuclear waste encapsulation, optics, and fusion reactors.

Hydrogen-related and OH-containing defects also play an important role in amorphous silica films used in microelectronic devices. However, [4H]_*Si*_ defects are rarely discussed in this context because they are thermodynamically costly to create, and hence their concentration is thought to be low. However, silica glass samples and amorphous films contain a lot of hydrogen introduced by the glass production and film deposition processes that often involve water and result in various defects that are difficult to detect [21,22]. Hydrogen-related defects are known to be strongly involved in reliability issues in microelectronic devices, such as the negative bias temperature instability (NBTI) [23,24] induced by hole injection from electrodes. Therefore, our findings can also be useful for understanding these phenomena.

In this study, we used DFT with a non-local functional to calculate the structure and properties of [4H]_*Si*_ defects and model their reactions with electron holes and hydrogen species in quartz. These species can be introduced into quartz or amorphous silica as a result of irradiation or injected from electrodes in devices. We studied α-quartz as a prototype SiO_2_ structure to provide a more detailed description of fully and partially H-passivated Si vacancies, as well as to predict the charge transition levels of these defects and their passivation and depassivation channels in the presence of excess atomic hydrogen. The reactions of holes and hydrogen with [4H]_*Si*_ can lead to the removal of the hydrogen atom from one or several silanol groups inside the [4H]_*Si*_ defect. This results in the formation of Si–O· dangling bonds, which are known as non-bridging oxygen hole centres (NBOHCs). This mechanism of formation of NBOHCs has not been discussed in the literature.

NBOHCs are common in silica glass and are characterised by several absorption bands and an emission band at 1.9 eV [25]. It has been demonstrated that these defects can be created in crystalline α-quartz by 2.5 MeV electrons and by fast neutrons [26]. Recent results demonstrate that, at low temperatures, NBOHCs in amorphous SiO_2_ are created as a result of electronic excitation and may be produced either through the O-H bond-scission in silanol groups (from OH impurities) or through the decay of strained Si-O bonds associated with self-trapped excitons [27]. We modelled how hole trapping by [4H]_*Si*_ defects may result in the formation of NBOHCs as a part of [3H]*_Si_* and calculated the optical absorption spectra of these centres.

The results demonstrate that atomic hydrogen in positive, neutral, and negative charge states has similar migration barriers of about 0.3 eV but different paths in pristine quartz. The trapping of electron holes by [4H]_*Si*_ defects and reactions with H^0^ leads to the breaking of O-H bonds and the formation of NBOHCs within the depassivated defects. The calculated optical absorption energies of these centres are close to those attributed to individual NBOHCs in glass samples. Although fully passivated [4H]*_Si_* defects are electrically inert, the removal of at least two hydrogen atoms from the defect makes them electrically active with respect to the trapping of holes from electrodes in devices.

## 2. Methods of Calculations

**Figure 1:**
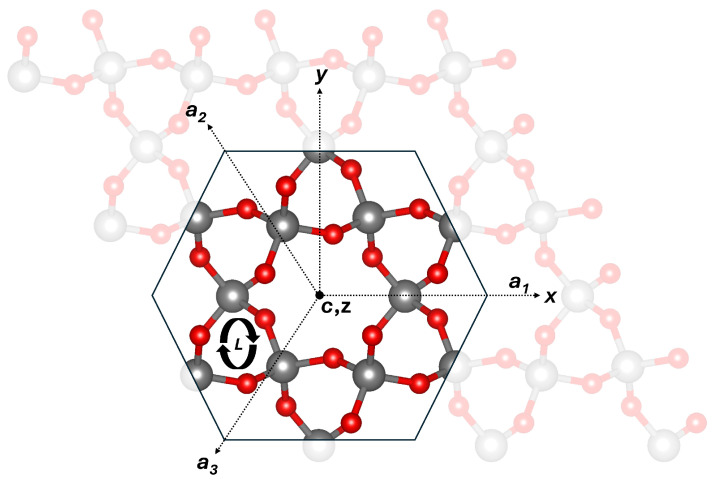
Projection of the 243-atom right-handed α-quartz structure onto the (0001) plane, perpendicular to the c-axis. This figure illustrates the EPR coordinate system (xyz) as well as crystallographic axes a_1_, a_2_, and a_3_, with the z-axis parallel to the c-axis. The central c-axis channel is surrounded by six smaller c-axis channels. Left-handed helices (L) are indicated. Gray and red spheres represent silicon and oxygen atoms, respectively.

All DFT calculations were performed using a 3 × 3 × 3 supercell of a right-handed α-quartz structure containing 243 atoms, as shown in Figure 1. The right-hand enantiomorph is defined by the arrangement of SiO_4_ tetrahedra in helical chains. These helices spiral in opposite directions along the crystal’s c-axis. In right-handed quartz, the helix twists clockwise when viewed along the c-axis [1].

The Gaussian plane wave (GPW) method implemented in the CP2K (v2024.3) package [28] was used in all calculations. The real-space integration plane-wave cutoff was converged to 650 Ry with a relative cutoff of 70 Ry. We employed a triple-ζ basis set with a set of polarisation functions in conjunction with the Goedecker–Teter–Hutter (GTH) pseudopotentials [29]. The non-local hybrid functional PBE0_TC_LRC [30] with a 25% Hartree–Fock exchange and an exchange truncation radius of 2.0 Å was used in all calculations to accurately represent the electronic characteristics of crystalline SiO_2_. The auxiliary density matrix method (ADMM) with the pFIT3 auxiliary basis set was used for Si, O, and H in addition to the current basis sets to minimise the computational cost of the Hartree–Fock exchange calculation [31].

The total energy of the system was converged to an accuracy of 1.0 × 10^−6^ eV using a combination of orbital transformation (OT) and Broyden mixing methods for diagonalisation of the Kohn–Sham matrix. The Broyden–Fletcher–Goldfarb–Shanno (BFGS) method was used to minimise the atomic forces to within 37 pN (0.023 eV Å^−1^). The cell vectors in silicon vacancy and hydrogen-related calculations were kept fixed at their initial α-quartz cell values.

The Kohn–Sham (KS) bandgap of defect-free α-quartz is 8.3 eV, which is underestimated compared to the experimental values reported in early studies by Bart et al., ranging from 8.6 to 9.5 eV [32,33] and 9.6 eV by Garvie et al. [34,35].

The diffusion barriers for H^+^, H^0^, and H^−^ were calculated using the climbing image nudged elastic band method (CI-NEB) [36]. Seven replicas were used between the initial and final configurations, determined via linear interpolation. The images were connected by springs with a force constant of 2 eV Å^−2^. The lowest diffusion barrier was obtained from four different positions for each hydrogen charge state.

Optical absorption spectra were calculated using linear-response time-dependent density functional theory (LR-TDDFT) implemented in CP2K [28]. This approach provides excitation energies within the Tamm–Dancoff approximation (TDA). For all optical absorptions, 1000 excitation energies were computed, employing a threshold of $1\times 10^{-4}$ eV for the convergence of the Davidson algorithm.

The defect formation energies were calculated using the standard relation:

(1) $$E_{form}=E_{def}-E_{bulk}-\Sigma \mu +q\left(E_{VBM}+E_{F}\right)+E_{corr},$$

where $E_{def}$ is the total energy of the defective system, $E_{bulk}$ is the energy of the pristine quartz structure, and Σμ is the sum of the chemical potentials. The chemical potentials for hydrogen and silicon were taken as the energies of a single hydrogen atom in the gas phase and single silicon atom from bulk silicon, respectively. $E_{F}$ is the Fermi energy of the system, *q* is the charge state of the defect, and $E_{VMB}$ is the energy of the valence band maximum in the defect-free system. $E_{corr}$ is the correction term for the periodic interaction between localised charges in charged systems calculated using the method proposed by Lany and Zunger [37].

Finally, we note that some of the reactions considered here lead to defect configurations that may have different spin states. Although all of them were considered, we describe only the lowest energy configurations.

## 3. Results and Discussion

Hydrogen is a common impurity in natural and synthetic quartz, introduced during crystallisation under hydrothermal conditions or subsequent high-temperature geological processes. Within the α-quartz lattice, hydrogen can occupy interstitial sites and bind to oxygen atoms to form silanol groups (Si–OH) [5]. It can also exist as neutral atomic hydrogen (H^0^) or protons (H^+^). At high Fermi-level positions, such as in microelectronic components, H^−^ ions can also be formed [38].

Understanding the mechanisms of diffusion of interstitial hydrogen is important because it governs the kinetics of various processes within the lattice. Hydrogen diffusion is determined by the structure of the SiO_2_ lattice, with pathways defined by interconnected silicon–oxygen rings and channels that determine energy barriers for diffusion depending on the size of the channel, steric hindrance, and the local defect structure. Due to various diffusion pathways and SiO_2_ morphology, a wide range of diffusion barriers between 0.15 and 2.04 eV have been reported [39,40,41,42,43,44,45,46,47]. To verify the previous predictions, we carried out calculations for the diffusion barriers of hydrogen in different charge states in perfect quartz.

### 3.1. Barriers to Hydrogen Diffusion in Different Charge States

**Figure 2:**
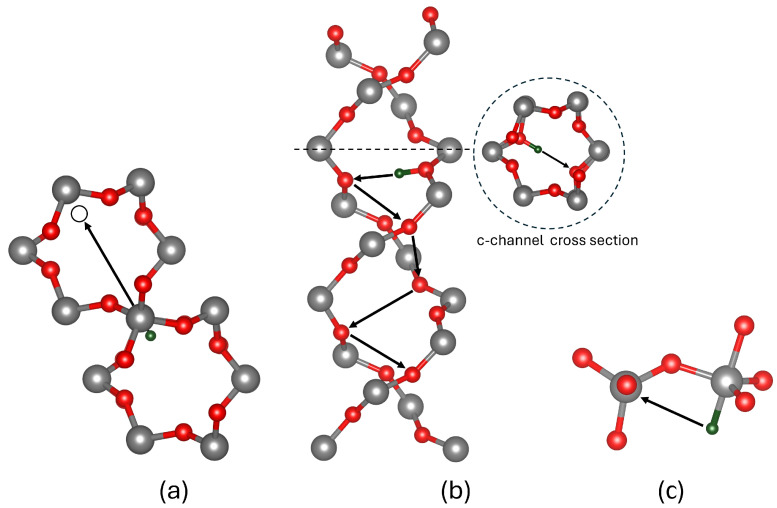
Hydrogen migration trajectories in α-quartz for (**a**) neutral charge state, illustrating migration across adjacent c-channels; (**b**) positive charge state, revealing the helical structure and cross-sectional view of c-channels; and (**c**) negative charge state, showing hopping along neighbouring Si tetrahedra. Arrows indicate the direction of motion. Gray, red, and green spheres represent silicon, oxygen and hydrogen atoms, respectively.

The migration of neutral atomic hydrogen (H^0^) was investigated within the large c-channel, between parallel c-channels, and via smaller c-channels connecting parallel c-channels, as seen in Figure 1. The lowest energy barrier of 0.34 eV corresponds to migration between parallel large c-channels connected by a SiO_4_ tetrahedron, as shown in Figure 2a. Surprisingly, migration along the large c-channel itself has a higher barrier of 1.0 eV. We note that due to its very small mass, the zero-point energy (ZPE) of hydrogen is important. The ZPE of 0.06 eV reported by Tuttle [48] lowers the H^0^ barrier to 0.28 eV, in closer agreement with the data reported by Griscom [40] of 0.18 eV and Kajihara [41] of 0.2 eV for glassy SiO_2_.

**Figure 3:**
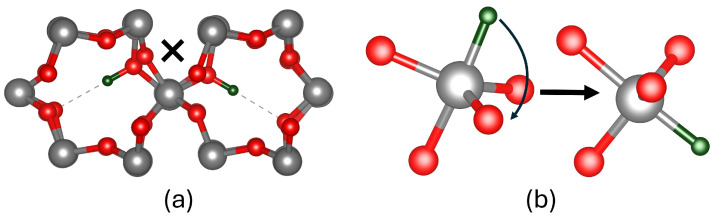
Rotation trajectories in α-quartz: (**a**) proton trajectory, with ‘**X**’ marking the transition state where the proton hops to a bridging oxygen within the same tetrahedron before rotating inward toward the c-channel; (**b**) hydride ion trajectory shown by the arrow, demonstrating rotation within the same tetrahedron and in between oxygen atoms in the same plane, resulting in the widening of the O–Si–O bond angle. Gray, red, and green spheres represent silicon, oxygen and hydrogen atoms, respectively.

In contrast, protons (H^+^) are bound to two-coordinated bridging oxygens within the SiO_2_ network. Several diffusion pathways for protons were investigated, including hopping to adjacent bridging oxygens, migration across rings, and migration along the c-channel. The lowest energy barrier of 0.29 eV corresponds to migration across a ring within a c-channel, as shown in Figure 2b. Thus, migration along a c-channel is the fastest. However, the barrier for the rotation of a proton between adjacent bridging oxygens within neighbouring c-channels was calculated to be 1.12 eV. The migration between adjacent bridging oxygens has a complex trajectory that involves rotation and switching between two O ions, as shown in Figure 3a. Experimental diffusion barriers have yet to be measured in quartz; however, the electric-field-induced transport of protons in amorphous SiO_2_ [49] suggests values of 0.38 eV and 0.8 eV in silica thin films [50]. Previous GGA calculations [51] predicted values of 0.5 eV for cross-ring migration barriers, close to our results obtained with a non-local functional in a bigger periodic cell.

Finally, we consider the H^−^ ion, which makes a bond with the Si ions in the lattice [38,52] and repels the O ions. As a result, O–Si–O angles in the tetrahedron open to form the trigonal bipyramidal structure shown in Figure 2c. There is a number of pathways for H^−^ migration in α-quartz including hopping between Si ion and rotations. The migration to a neighbouring Si atom along the c-channel requires overcoming the lowest energy barrier of 0.34 eV, as shown in Figure 2c. Unlike protons, H^−^ ions can rotate within the same silicon site with a barrier of 0.35 eV, as illustrated in Figure 3b. This rotational flexibility highlights a key distinction between the behaviours of the H^−^ and H^+^ ions in the SiO_2_ matrix. We are not aware of experimental or theoretical results on hydride diffusion.

### 3.2. Hole Trapping at Si Vacancy Centres

In silicon-deficient quartz systems, it is proposed that silicon vacancies are fully passivated by hydrogen, forming a [4H]*_Si_* or hydrogarnet defect, which has been experimentally identified in mineral studies [1,5,10]. The most energetically favourable configuration of the [4H]*_Si_* defect contains four silanol groups with average Si–O and O–H bond distances of 1.61 Å and 0.97 Å, respectively. The Si–O–H angle ranges between 118 and 121° to promote hydrogen bonding between the O–H groups.

**Figure 4:**
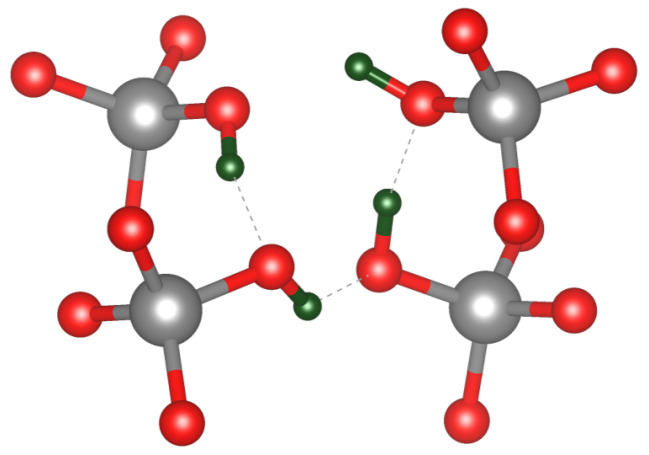
Fully passivated silicon vacancy [4H]_*Si*_ defect in α-quartz. Dashed lines indicate the hydrogen bonding interactions within the vacancy centre. Gray, red, and green spheres represent silicon, oxygen and hydrogen atoms, respectively.

We begin by considering a fully passivated [4H]*_Si_* defect using a pristine α-quartz model. The lowest energy configuration aligns with the structure described in [10] where three silanol groups are oriented toward the silicon vacancy centre and one facing out, as can be seen in Figure 4. In contrast, earlier theoretical studies of these defects found silanol groups facing away from the vacancy [11]. We note that rotations of the O-H bonds may introduce a range of structural variations. However, energy differences of approximately 0.2 eV when rotating a singular O-H group away from the vacancy centre lead to 0.2 eV barriers, suggesting that fast, free rotation of the O-H bond at room temperature is unlikely.

**Figure 5:**
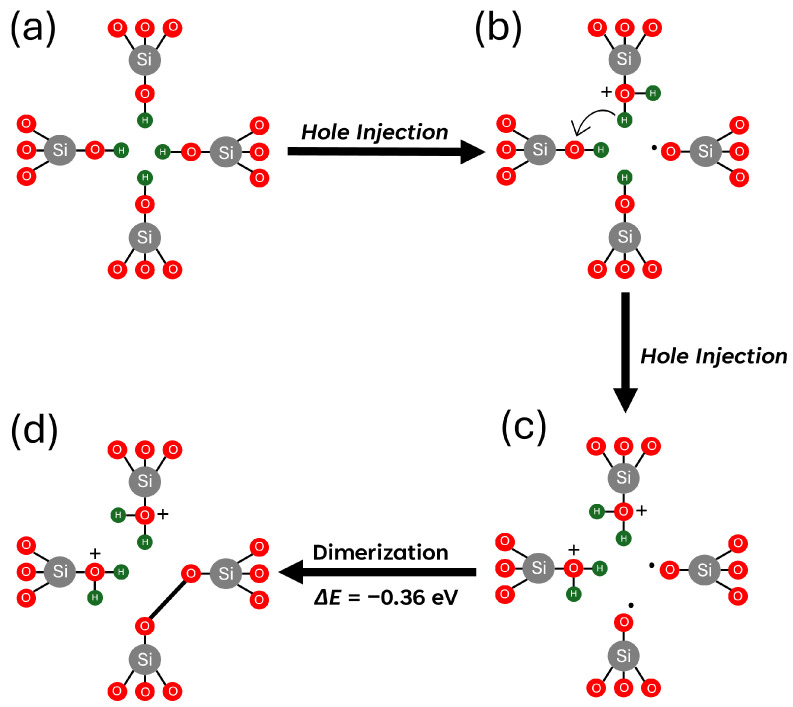
Schematic representation of the sequential hole-trapping mechanism at a [4H]_*Si*_ defect in α-quartz. (**a**) Neutral, fully passivated state. (**b**) Trapping of the first hole, forming a singly positive defect. (**c**) Trapping of the second hole, resulting in a doubly positive defect (**d**) Structural dimerization of the doubly positive [4H]_*Si*_ defect. Gray, red, and green spheres represent silicon, oxygen, and hydrogen atoms, respectively.

Upon irradiation, holes are introduced into the system and can be trapped by the passivated silicon vacancy. The hole trapping triggers a spontaneous migration of a proton and binding to an adjacent oxygen atom in an O–H bond, as shown in Figure 5b, increasing the Si–O distance to 1.71 Å. This reaction results in the formation of a dangling 1.68 Å long Si-O bond with an unpaired electron on the terminal oxygen, also known as the NBOHC [53]. This result differs from the model proposed in [17], where the hole is initially trapped on an oxygen p orbital associated with one of the hydrogens. A proton subsequently diffuses away, leaving the NBOHC. Thus, hole trapping can lead to effective depassivation of the Si vacancy by reducing the number of hydrogen atoms bound to dangling Si–O bonds. However, the EPR data in [17] suggest that the proton separation from the remaining defect requires significant time or, in other words, overcoming an energy barrier.

Our proposed mechanism of the hole-induced depassivation of [4H]*_Si_* is shown schematically in Figure 5. Upon irradiation, a hole introduced into the system is trapped in the [4H]*_Si_* defect (Figure 5a), triggering a spontaneous dissociation of the proton into a nearby silanol group that forms an entity –OH_2_^+^ that bears the positive charge (Figure 5b). However, a barrier of 1.35 eV is required to move the proton to a bridging two-coordinated oxygen ion nearby and further away from the defect, consistent with the observations in [17]. The proton can hop between different Si–O–H groups inside the Si vacancy with a small barrier of about 0.03 eV. This mechanism can create NBOHCs perturbed by a proton in irradiated samples containing [4H]*_Si_* defects.

The subsequent capture of the second hole through the mechanism described above creates a stable configuration with two protons and an O–O peroxy bridge inside the vacancy, as shown in Figure 5d. However, further depassivation is inhibited after the second hydrogen is removed, as hole trapping occurs preferentially at the peroxy bridge. This process leads to a reduction in the O–O bond length from 1.45 Å to 1.30 Å, with the trapped hole delocalised across both O p orbitals.

### 3.3. Hydrogen Reactions with Si Vacancy Centres

**Figure 6:**
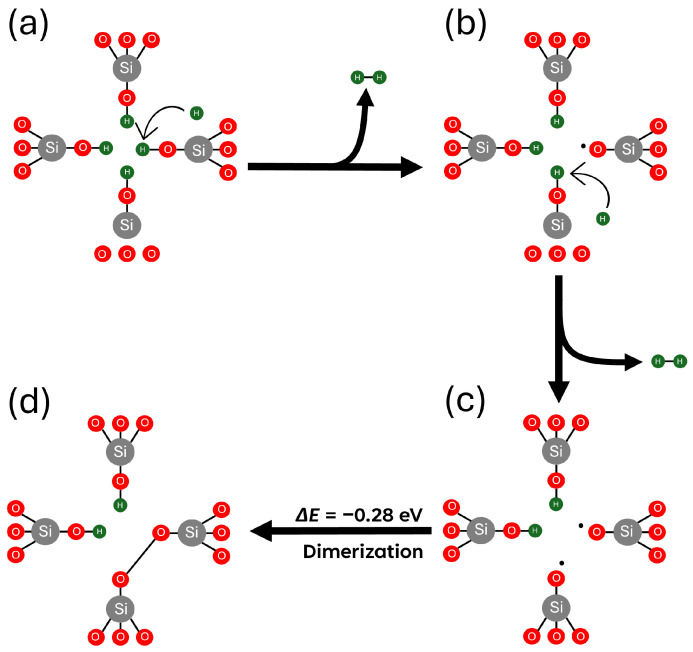
Schematic representation of H^0^-assisted depassivation of a [4H]_*Si*_ defect in α-quartz. (**a**) Neutral, fully passivated state interacting with H^0^. (**b**) Singly depassivated state interacting with a second H^0^ atom. (**c**) Formation of a defect with two hydrogens removed, exposing two NBOHCs. (**d**) Dimerization of the doubly depassivated [4H]_*Si*_ defect. Gray, red, and green spheres represent silicon, oxygen and hydrogen atoms, respectively.

Hydrogen dissociation from a [4H]*_Si_* defect in α-quartz is thermodynamically unfavourable, requiring about 6.0 eV to extract a hydrogen atom and position it interstitially, as described in reaction (i) in Table 1. However, silica samples are often subjected to high hydrogen concentrations at elevated temperatures, such as quartz at high pressures within the Earth’s crust and amorphous silica under electrical bias in SiO_2_-based microelectronic devices. These conditions make [4H]*_Si_* defects susceptible to reactions with hydrogen. However, the energy required to remove one of the passivating H atoms from [4H]*_Si_* as a result of a reaction with an extra H atom is 1.04 eV with a reaction barrier of 1.2 eV at 0 K, as shown in reaction (ii) in Table 1. This reaction, producing molecular hydrogen (H_2_), can occur only at elevated temperatures. The reaction with the second H atom reduces the number of NBOHC defects and facilitates the formation of an O–O peroxide bridge, as shown in Figure 6. However, the reverse reaction of atomic H has a small barrier, and a peroxide bridge exhibits high reactivity toward atomic H, leading to breaking of a single O–O bond and forming an O-H bond with an energy gain of 5.29 eV. Therefore, the depassivation of [4H]*_Si_* is unlikely under high excess neutral atomic H.

Atomic hydrogen in silica can exist in multiple charge states depending on environmental conditions and the processes involved. Under irradiation, the generation of electron–hole pairs enables the trapping of holes by hydrogen atoms, forming protons (H^+^) in systems where the Fermi level lies below 5 eV below the conduction band minimum (CBM) [38]. When H^+^ approaches the vacancy, it preferentially binds to a nearby O-H group instead of depassivating the vacancy. This binding results in the most energetically favourable configuration, with a formation energy of −0.61 eV. This interaction, referred to as reaction (iii), represents a stable proton–vacancy complex.

**Figure 7:**
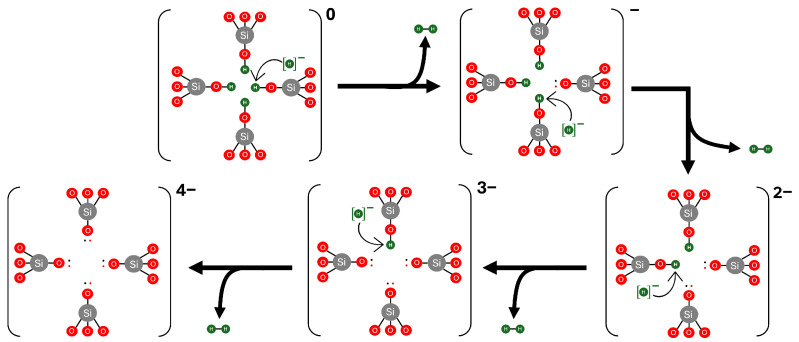
Schematic representation of the reaction of hydride ions (H^−^) with a [4H]_*Si*_ defect in α-quartz. The reaction leads to the depassivation of the defect and the subsequent formation of molecular hydrogen (H_2_). Gray, red, and green spheres represent silicon, oxygen and hydrogen atoms, respectively.

In contrast, when a H^−^ ion is formed, we observe a spontaneous depassivation mechanism facilitated by H^−^. This process results in the formation of a H_2_ molecule and a Si–O^−^ dangling bond, as described by reaction (iv) in Table 1. This reaction is thermodynamically favourable, with a formation energy of −0.98 eV for the first depassivation event. In particular, this pathway does not require an activation barrier for hydrogen depassivation or dissociation, making it the most likely mechanism for the continuous hydrogen depassivation of [4H]*_Si_*. The subsequent depassivation steps are illustrated in Figure 7.

### 3.4. Charge Transition Levels of [4H]_Si_ Defects

**Figure 8:**
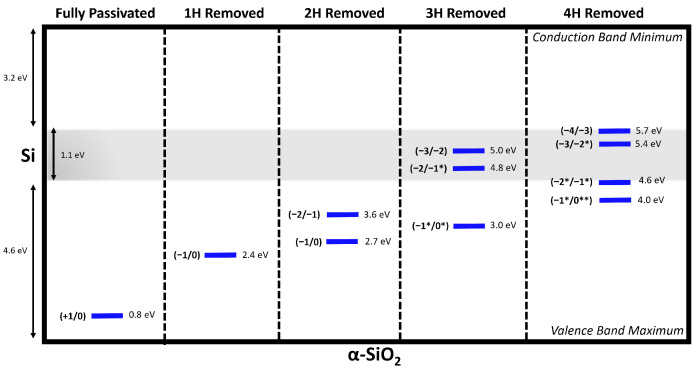
Band diagram of the Si/SiO_2_ interface showing the hole-trapping charge transition levels (blue) of a [4H]_*Si*_ defect and the negatively charged states of the semi- and fully depassivated silicon vacancies. Single (*) and double (**) asterisks denotethe charge states with single and double dimer configurations, respectively. The band alignment assumes an average valence band maximum (VBM) offset of 4.6 eV based on spectroscopy experiments [55,56,57], with bandgaps of 1.1 eV (gray shaded area) and 8.9 eV for SiO_2_, respectively.

The reactions described above are particularly relevant in microelectronic device applications, where the position of the Fermi level inside the oxide film is determined by the band offset with the electrodes and the bias between electrodes. Device operation conditions often promote high Fermi levels. Under these conditions, electrons or holes can be injected into the oxide from the electrodes and trapped at defects, changing their charge states. The charge transition level (q/q′) of a defect is the Fermi energy at which the formation energies of charge states q and q′ are equal. Under these conditions, H^−^ has been shown to be stable at Fermi levels exceeding approximately 3 eV in α-cristobalite [54] and 4 eV below the bottom of the conduction band in α-quartz [38]. In Si/SiO_2_-based devices, such as metal–oxide semiconductor field-effect transistors (MOSFETs), where the Fermi level lies at around 5 eV below the CBM of SiO_2_, p-type MOSFETs are dominated by hole carriers at the top of the Si valence band. As can be seen in Figure 8, H^−^-depassivated silicon vacancies introduce charge transition levels (CTLs) that resonate with the Si valence band. This may enable hole tunneling into defect states, particularly in the case where 3 or 4 hydrogens are removed, reducing their charge. These defects may contribute to leakage current and device degradation.

### 3.5. Optical Absorption of NBOHC in [4H]_Si_

Previous experimental measurements [25,26] and calculations for NBOHCs have been carried out on the (001) surface of α-quartz or in bulk SiO_2_ glass structures. They are not directly related to passivated silicon vacancies, where the local geometry and interactions with other silanol groups may affect the optical transitions. Therefore, it is of interest to calculate the optical transitions in NBOHCs embedded in a hydrogarnet defect.

**Figure 9:**
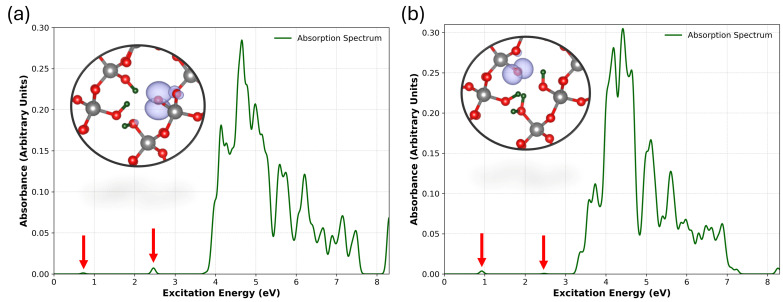
Optical absorption spectra of a [4H]_*Si*_ defect for (**a**) the neutral charge state with one hydrogen removed and (**b**) a hole-trapped [4H]_*Si*_ defect. Red arrows indicate the position of the excitation energies with low oscillator strengths (the oscillator strength is normalised, with Gaussian smearing applied using σ=0.05). Insets depict atomic structures, with the unoccupied p orbital shown in purple. Gray, red, and green spheres represent silicon, oxygen and hydrogen atoms, respectively.

We compare the optical absorption spectra for the NBOHCs generated by H^0^-assisted depassivation at elevated temperatures and low hydrogen concentration with the NBOHCs generated as a result of hole trapping by [4H]_*Si*_ followed by spontaneous H^+^ dissociation in Figure 9a,b. Previous studies of NBOHCs in SiO_2_ have reported three optical absorption bands associated with isolated NBOHCs: a band at 2.0 eV, a broader band in the UV range above 3.8 eV with the maximum at about 4.8 eV, and a further peak at ≈6.8 eV [58]. A very weak transition at ≈0.2 eV is associated with electron excitation between partially occupied *p* states on the dangling O ion [58].

In the neutral case with one hydrogen removed, the lowest energy weak transition has an energy *T_e_* of 0.72 eV and an oscillator strength *f* = $6.8\times 10^{-5}$. This $T_{e}$ value is higher than those reported in previous studies: 0.52 eV (*f* = $1.0\times 10^{-4}$) by Giordano et al. [59], 0.24 eV (*f* = $7.7\times 10^{-6}$) by Pacchioni [60], and 0.20 eV (*f* = $3.0\times 10^{-6}$) by Suzuki et al. [58]. This transition has been attributed to electronic excitations between the doubly and singly occupied nonbonding O-2p_*y*_ and O-2p_*x*_ orbitals of the NBOHC [59]. The observed excited state comprises 64 individual transitions, with the highest excitation amplitude arising from transitions involving delocalised nonbonding oxygen p orbitals located below the valence band maximum. The presence of a proton within the defect, as shown in Figure 5, results in a *T_e_* = 0.92 eV (*f* = $2.0\times 10^{-4}$) transition, indicating a 0.2 eV shift relative to the neutral configuration, accompanied by an increase in the oscillator strength. The larger absorption energy along the series reflects the lowering of the local symmetry around the defect for the two defects, which increases the energy difference between the two nonbonding orbitals of the terminal oxygen [59].

The next notable absorption peak was observed at *T_e_* = 2.46 eV (*f* = $3.9\times 10^{-4}$). In comparison, Ref. [59] reported *T_e_* = 2.19 eV (*f* = $6.0\times 10^{-4}$), while Ref. [60] and Ref. [58] reported T*_e_* = 2.02 eV (*f* = $5.6\times 10^{-4}$) and *T_e_* = 2.02 eV (*f* = $2.5\times 10^{-5}$), respectively. This peak was initially assigned to the electron transitions between the nonbonding orbitals of the oxygen ions nearest to the NBOHC and the O_*nb*_-2p_*y*_ orbital. However, Giordano et al. [59] later attributed it primarily to transitions from a σ-bonding orbital, predominantly with O_*nb*_-2p*_z_* characteristics, to the singly occupied O-2p_*y*_ orbital of the NBOHC. This attribution is consistent with the transition observed in this study. Furthermore, we report that the σ-bonding p-orbital lies 3.9 eV below the valence band maximum, consistent with the findings of Ref. [59]. In the presence of the proton, this transition is observed at 2.47 eV (*f* = $3.7\times 10^{-5}$) with a minor shift of 0.1 eV. This indicates the influence of the positive charge on the transitions involving the oxygen p orbitals of the NBOHC.

The UV broad band with an onset at about 3.8 eV has several components, as seen in Figure 9. These peaks initiate at *T_e_* = 3.77 eV (*f* = $1.0\times 10^{-4}$) and slowly drop to approximately 7.5 eV. Taking into account the peaks with the highest oscillator strengths, we report the following *T_e_*: 3.97, 4.11, 4.55, 4.6 (highest oscillator strength), 4.77, and 5.15 eV. Giordano [59] identified three significant peaks in α-quartz (Gaussian smearing, σ = 0.2), located at *T_e_* = 3.5, 4.1, and 4.7 eV, with the silanol group contributing to the peak at 3.5 eV. We report the silanol contribution at a transition energy of 3.7 eV. This band is similar to the broad band at 4.8 eV in silica glass [25]. In the presence of the proton, narrower peaks emerge above 3.74 eV, no longer associated with the transitions between silanol groups; instead, they involve bonding oxygen p-states below the valence band. The strongest transition was identified at 4.45 eV (*f* = $5.8\times 10^{-3}$), accompanied by smaller peaks at higher energies, including a notable transition at 6.64 eV (*f* = $1.2\times 10^{-3}$). These transitions predominantly involve occupied O-2p states located below the valence band.

Finally, we observe a number of weaker peaks around 6.0–7.5 eV, attributed to transitions from the valence band bonding O-p states to the nonbonding O-2p states of the NBOHC in α-quartz. Similar transitions were reported around 6 eV [59] and 6.2 eV [58].

The calculated spectrum closely resembles the experimental spectra observed in silica glass and those calculated for the silica surface. However, some peaks are shifted because of the presence of silanol groups and an additional proton in the vicinity of the NBOHC within the Si vacancy. These results indicate that the shift in the sharp 2.0 eV peak serves as a reliable indicator of the perturbation induced by the environment surrounding the NBOHC.

## 4. Conclusions

We used hybrid density functional theory to investigate the structure and properties of [4H]_*Si*_ defects in α-quartz, and the reactions of these defects with electron holes as well as extra hydrogen atoms and ions. Initially, we examined the mechanisms of hydrogen migration in various charge states within pure quartz. The results indicate low migration barriers for H^+^, H^0^, and H^−^, involving distinct pathways through c-channels.

The reaction of electron holes with [4H]_*Si*_ induces a structural transformation of defects, wherein the hole becomes trapped on a proton that detaches from its original site and attaches to an oxygen ion of an adjacent Si-O-H group within the vacancy. This is accompanied by the formation of NBOHCs inside the vacancy. However, the energy barrier for the proton to migrate away from the defect is quite high at 1.35 eV, suggesting that this should be a slow process, in agreement with experimental observations in ref. [17].

Extra protons strongly bind to [4H]_*Si*_ defects, producing positively charged centres. The reaction of hydrogen atoms with [4H]_*Si*_ can lead to the creation of H_2_ molecules and NBOHCs; however, this reaction is endothermic and requires overcoming a barrier of 1.2 eV. Small barriers for reverse reactions suggest that this process is only feasible when the amount of excess hydrogen atoms is much lower than that of [4H]_*Si*_ defects, which is quite unlikely. On the other hand, H^−^ ions react strongly with [4H]_*Si*_, producing H_2_ and negatively charged defects. These defects can trap holes if they are injected from the electrodes in devices.

We note that the recent work by Skuja et al. [26] demonstrated the formation of NBOHCs in electron-irradiated crystalline quartz, which did not undergo amorphisation. The authors speculated that the formation of NBOHCs in $e^{-}$-irradiated quartz indicates the formation of internal voids of size comparable to that of the voids in glassy SiO_2_. Our results demonstrate that the reactions of electron holes and H^0^ with [4H]_*Si*_ defects can also result in the breaking of O–H bonds and the formation of NBOHCs. This mechanism of formation of NBOHCs in silica has not been considered in the literature. The calculated optical absorption spectra of NBOHCs align with the experimental data and prior calculations, showing shifts in the positions of the characteristic peaks due to the interactions with the Si–O–H groups within the Si vacancy. Specifically, the shift in the sharp 2.0 eV peak can serve as an indicator of the perturbation induced by the NBOHC environment.

These findings offer insights into the structure and reactions of [4H]_*Si*_ defects in quartz and silica and the formation and evolution of NBOHCs under irradiation. They can be used to better understand the radiation-induced defect processes in quartz and radiation damage of silica glasses used in nuclear waste encapsulation, optics, and fusion reactors. [4H]_*Si*_ defects are much less known in the context of microelectronic devices, and our results suggest that they can be transformed into electrically active defects under the conditions common in devices.

## Figures and Tables

**Table 1:** Hydrogen related reactions with passivated silicon vacancy centres in α-quartz.

id	Reaction Mechanism	ΔE (eV)
i	Si(OH)_3_-OH → Si(OH)_3_-O^·^ + H^0^	+6.0
ii	Si(OH)_3_-OH + H^0^ → Si(OH)_3_-O^·^ + H_2_	+1.04
iii	Si(OH)_3_-OH + H^+^ → Si(OH)_3_-${\mathrm{OH}}_{2}^{+}$	−0.61
iv	Si(OH)_3_-OH + H^−^ → Si(OH)_3_-O^−^ + H_2_	−0.98

## Data Availability

The data required to reproduce these findings are available from the corresponding author upon reasonable request.

## Abbreviations

The following abbreviations are used in this manuscript:

NBOHC	Non-bridging oxygen hole center
DFT	Density functional theory
EPR	Electron paramagnetic resonance
NBTI	Negative bias temperature instability
GPW	Gaussian plane wave
GTH	Goedecker–Teter–Hutter
ADMM	Auxiliary density matrix method
OT	Orbital transformation
BFGS	Broyden–Fletcher–Goldfarb–Shanno
KS	Kohn–Sham
CI-NEB	Climbing image-nudged elastic band
LR-TDDFT	Linear-response time-dependent density functional theory
TDA	Tamm–Dancoff approximation
ZPE	Zero-point energy
CBM	Conduction band minimum
MOSFET	Metal–oxide semiconductor field-effect transistor
CTL	Charge transition level
VBM	Valence band maximum
